# Old age and *EGFR* mutation status in inoperable early‐stage non‐small cell lung cancer patients receiving stereotactic ablative radiotherapy: A single institute experience of 71 patients in Taiwan

**DOI:** 10.1111/1759-7714.14786

**Published:** 2023-01-18

**Authors:** Yuan‐Hung Wu, Yu‐Mei Kang, Yu‐Wen Hu, Keng‐Li Lan, Sang‐Hue Yen, Tzu‐Yu Lai, Tien‐Li Lan, Yuh‐Min Chen, Chao‐Hua Chiu, Yung‐Hung Luo, Heng‐sheng Chao, Chi‐Lu Chiang, Tsu‐Hui Shiao, Chao‐Neng Yang, Wen‐Hu Hsu, Yu‐Chung Wu, Han‐Shui Hsu, Jung‐Jyh Hung, Chien‐Sheng Huang, Po‐Kuei Hsu, Yi‐Wei Chen

**Affiliations:** ^1^ Department of Oncology Taipei Veterans General Hospital Taipei Taiwan; ^2^ School of Medicine National Yang‐Ming Chiao‐Tung University Taipei Taiwan; ^3^ Department of Biomedical Imaging and Radiological Sciences National Yang‐Ming Chiao‐Tung University Taipei Taiwan; ^4^ Institute of Traditional Medicine National Yang‐Ming Chiao‐Tung University Taipei Taiwan; ^5^ Department of Radiation Oncology Taipei Municipal Wan‐Fang Hospital Taipei Taiwan; ^6^ Department of Chest Medicine Taipei Veterans General Hospital Taipei Taiwan; ^7^ Taipei Cancer Center and Taipei Medical University Hospital Taipei Medical University Taipei Taiwan; ^8^ Department of Surgery Taipei Veterans General Hospital Taipei Taiwan; ^9^ Institute of Emergency and Critical Care Medicine National Yang‐Ming Chiao‐Tung University Hsinchu Taiwan

**Keywords:** EGFR, lung cancer, old age, SABR

## Abstract

**Background:**

Stereotactic ablative radiotherapy (SABR) is now the standard of care for patients with inoperable early‐stage lung cancer. Many of these patients are elderly. *EGFR* (epidermal growth factor receptor) mutation is also common in the Asian population.

**Methods:**

To evaluate the effects of old age and *EGFR* mutation on treatment outcomes and toxicity, we reviewed the medical records of 71 consecutive patients with inoperable early‐stage non‐small cell lung cancer (NSCLC) who received SABR at Taipei Veterans General Hospital between 2015 and 2021.

**Results:**

The study revealed that median age, follow‐up, Charlson comorbidity index, and ECOG score were 80 years, 2.48 years, 3, and 1, respectively. Of these patients, 37 (52.1%) were 80 years or older, and 50 (70.4%) and 21 (29.6%) had T1 and T2 diseases, respectively. *EGFR* mutation status was available for 33 (46.5%) patients, of whom 16 (51.5%) had a mutation. The overall survival rates at 1, 3, and 5 years were 97.2, 74.9, and 58.3%, respectively. The local control rate at 1, 3, and 5 years was 97.1, 92.5, and 92.5%, respectively. Using Cox proportional hazards regression we found that male sex was a risk factor for overall survival (*p* = 0.036, 95% CI: 1.118–26.188). Two patients had grade 2 pneumonitis, but no other grade 2 or higher toxicity was observed. We did not find any significant differences in treatment outcomes or toxicity between patients aged 80 or older and those with *EGFR* mutations in this cohort.

**Conclusion:**

These findings indicate that age and *EGFR* mutation status do not significantly affect the effectiveness or toxicity of SABR for patients with inoperable early‐stage NSCLC.

## Graphical abstract

We reviewed 71 consecutive early‐stage inoperable NSCLC patients who received SABR at our institute, and there were no significant differences in patients aged 80 or older, or with *EGFR* mutation.

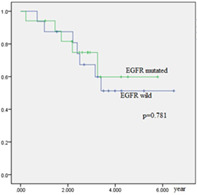

## INTRODUCTION

Lung cancer is a major global health burden, ranking as the second most common cancer worldwide with 2.2 million new cases in 2020. It is also the leading cause of cancer deaths, with approximately 1.8 million deaths in 2020.^1^ In Taiwan, lung cancer is the leading cause of cancer deaths, accounting for around 10 000 deaths each year.^2^ Effective screening tools are needed for the early diagnosis of lung cancer. Low‐dose computed tomography (LDCT) has been tested in smokers in randomized controlled trials (RCTs) and has shown survival benefits.^3^ LDCT has become increasingly popular in health examinations in the general population, even though the results of RCTs are still pending. This has led to an increase in the incidence of early‐stage lung cancer, as more cases of the disease are diagnosed at an early stage.^4^ The standard treatment for early‐stage lung cancer is surgical resection, typically a lobectomy, accompanied by radical lymph node dissection. For patients who are not candidates for surgery, radiotherapy is the standard alternative. Conventional fractionated external beam radiotherapy (EBRT) typically takes 5–7 weeks to complete. Stereotactic body radiotherapy (SBRT) or stereotactic ablative radiotherapy (SABR) offers higher precision and higher doses per fraction, allowing the entire course of treatment to be completed in 1–2 weeks. Randomized controlled trials have shown that SABR has lower toxicity compared to conventional EBRT, with similar or better treatment outcomes.^5^, ^6^ SABR has become the standard of care for inoperable patients with early‐stage lung cancer. Many of these patients are elderly, and it is of interest to determine whether there are differences in treatment outcomes for older patients. Taipei Veterans General Hospital (TVGH) is known for caring for one of the oldest populations in Taiwan. We reviewed the medical records of inoperable patients who received SABR at TVGH to determine if there were any differences in treatment outcomes for elderly patients. We also evaluated the presence of *EGFR* mutations in this population, as *EGFR* mutations are common in Asian patients with lung adenocarcinoma, particularly in nonsmoking female patients.^7^ The prognostic role of *EGFR* mutation status in early‐stage non‐small cell lung cancer (NSCLC) after surgery is still controversial.^8^, ^9^ Previous studies have examined the impact of *EGFR* mutations on treatment outcomes after SABR for early‐stage NSCLC, but the results have been mixed. Nakamura et al. found that patients with *EGFR* mutations had a similar local control rate, but a higher rate of out‐of‐field progression compared to patients without *EGFR* mutations, with five *EGFR*‐mutated patients in the study.^10^ Other studies have also examined the impact of *EGFR* mutations on treatment outcomes after SABR for early‐stage NSCLC. These studies, which included seven and 24 *EGFR*‐mutated patients, respectively, did not find a difference in treatment outcomes between patients with and without *EGFR* mutations.^11^, ^12^ However, the small sample sizes of these studies may be insufficient to draw definitive conclusions. Here, we analyzed the role of *EGFR* mutation and old age to determine their roles in prognosis and toxicity.

## METHODS

The cancer registry of Taipei Veterans General Hospital (TVGH) was reviewed using the following inclusion criteria: (1) aged 20 years or older, (2) pathological proof of non‐small cell lung cancer, (3) clinical stage cT1‐2N0M0, and (4) received SABR between 2015 and 2021 at TVGH. The exclusion criteria were: (1) tumors larger than 5 cm, and (2) distant metastasis of lung cancer or other synchronous cancer noted before SABR. The tumors were staged according to the AJCC seventh edition for patients diagnosed between 2015 and 2017, and according to the AJCC eighth edition for patients diagnosed in 2018 or later. Since this study only considered T1 or T2 stages and excluded tumors larger than 5 cm, the change in staging criteria did not affect the statistical analysis. IBM SPSS version 22 was used for statistical analysis. Associations between categorical and continuous variables were detected using two‐tailed Chi‐square tests and two‐tailed Student's *t*‐tests, respectively. Statistical significance between factors was determined using the log‐rank test. A *p*‐value less than 0.05 was considered statistically significant. The Cox proportional hazards model was applied to estimate hazard ratios and 95% confidence intervals (CIs). Toxicity was reported according to the Common Terminology Criteria for Adverse Events (CTCAE) 4.03. Survival duration was calculated from the last day of SABR. Progression‐free survival was defined as patients surviving without radiographic evidence of disease progression. Disease control was defined as patients without radiographic evidence of disease progression. Distant control was calculated until radiographic evidence of distant metastasis. Lung cancer‐specific survival was calculated until the patient died after radiographic evidence of lung cancer recurrence. Local control was calculated until radiographic evidence of local recurrence. The Charlson comorbidity index (CCI) was calculated according to the original definition without age‐adjustment.^13^

## RESULTS

### Patient characteristics

A total of 71 consecutive patients were reviewed. The demographics are shown below. The median age was 80 years old, median ECOG score was 1, median CCI score was 3, and median follow‐up time was 2.48 years. The patient characteristics are listed in Table 1.

**TABLE 1:** Patient characteristics

Characteristic	All *N* = 71	<80 y/o *N* = 34	≥80 y/o *N* = 37	*p*‐value
Age, median (range)	80 (42–93)	74.5 (42–79)	86 (80–93)	‐
Male	38 (53.5%)	17 (50%)	21 (56.8%)	0.57
Smoking	35 (49.3%)	16 (47.1%)	19 (51.4%)	0.718
ECOG (%)				0.926
0	6 (8.5%)	3 (8.8%)	3 (8.1%)	
1	47 (66.2%)	22 (64.7%)	25 (67.6%)	
2	15 (21.1%)	7 (20.6%)	8 (21.6%)	
3	3 (4.2%)	2 (5.9%)	1 (2.7%)	
*EGFR* mutation				0.401
Mutated	17 (23.9%)	7	10	
Wild	16 (22.5%)	6	10	
Unknown	38 (53.5%)	21	17	
Pathology				0.401
Adenocarcinoma	57 (80.3%)	27	30	
Mucinous adenocarcinoma	2 (2.8%)	0	2	
Adenosquamous	1 (1.4%)	1	0	
SqCC	10 (14.1%)	5	5	
NSCLC	1 (1.4%)	1	0	
Charlson comorbidity index				0.067
2	17	4	13	
3	26	13	13	
4	9	4	5	
5	8	6	2	
6	6	5	1	
7	3	2	1	
8	2	0	2	
Synchronous other cancer	10	5	5	0.885
NSCLC diagnosed in past 5 years	18	9	9	0.836
T stage				0.623
1	50	23	27	
2	21	11	10	

Abbreviations: ECOG, Eastern Cooperative Oncology Group Performance Status; EGFR, epidermal growth factor receptor status; SqCC, squamous cell carcinoma; NSCLC, non‐small cell lung cancer.

The dose‐fractionation of SABR performed is shown in Table 2. A total of 67 (94.3%) patients received a biological equivalent dose with α/β = 10(BED_10_) ≧ 100 Gy. More than 60% of the patients received 50 Gy in five fractions.

**TABLE 2:** Radiation dose, fraction number, and biologically equivalent dose

Total physical dose (Gy)	Fraction number	BED_10_ (Gy)	Number	(%)
45	6	78.8 Gy	1	1.4%
45	5	85.5 Gy	1	1.4%
48	6	86.4 Gy	2	2.8%
50	5	100 Gy	43	60.6%
53.1	6	100.1 Gy	4	5.6%
54	6	102.6 Gy	4	5.6%
55	5	115.5 Gy	2	2.8%
60	6	120 Gy	6	8.5%
34	1	149.6 Gy	8	11.3%

Abbreviations: BED, biologically effective dose; Gy, Gray.

### Treatment results

Among the patients who suffered from disease failure, six (8.5%) patients had local failure, two (2.8%) patients had regional failure, and 10 (14.1%) patients had distant failure.

Data on the *EGFR* mutation status was available for 33 (46.4%) patients. Among them, 17 (51.5%) patients were found to have *EGFR* mutation. The pattern of mutation is listed in Table 3. L858R (41.2%) and exon 19 deletion (35.3%) were the most common mutation sites.

**TABLE 3:** *EGFR* mutation status

Mutation site	Patient number (%)
Exon19 deletion	6 (35.3%)
L858R	7 (41.2%)
L861Q	2 (11.8%)
G719X	2 (11.8%)

**FIGURE 1:**
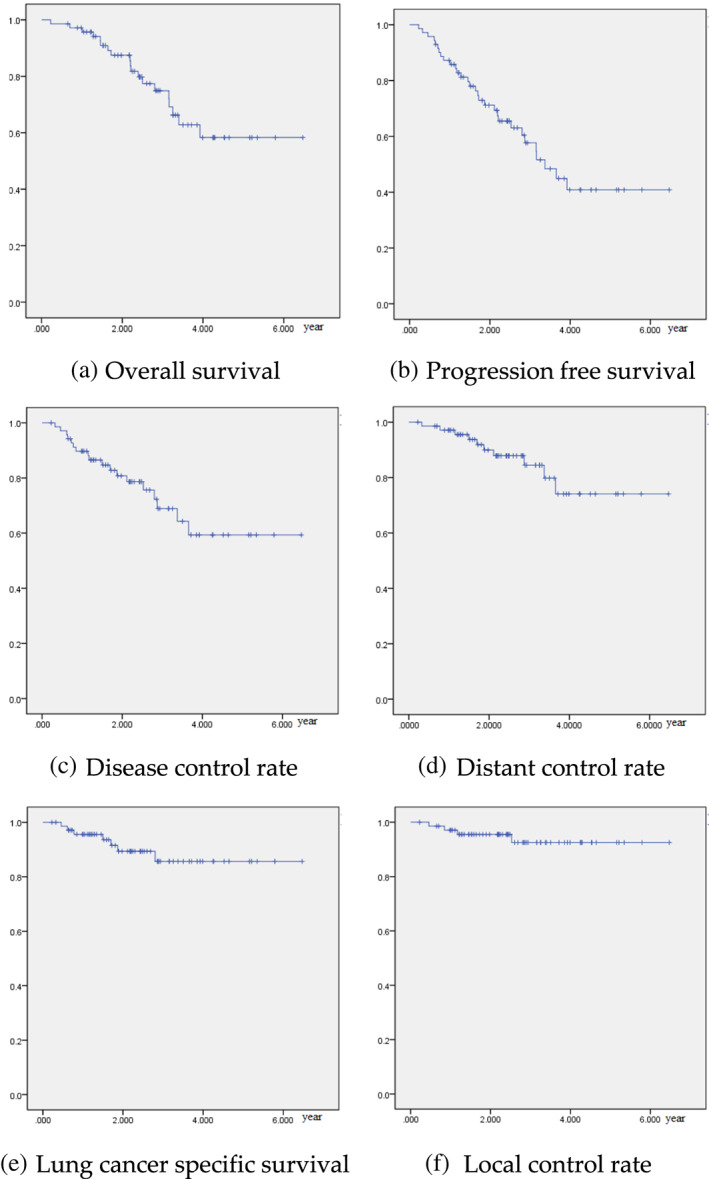
(a) Overall survival, (b) progression‐free survival, (c) disease control rate, (d) distant control rate, (e) lung cancer specific survival and (f) local control rate

Overall survival at 1, 3, and 5 years was 97.2%, 74.9%, and 58.3%. There were 29 patients who died during follow‐up. Of these, 17 patients were noted to have lung cancer progression before death and were determined to have died of the disease (Figure 1). Noncancer death was then considered for the other 12 patients (41.4%). Progression‐free survival at 1, 3, and 5 years was 87.2, 57.7, and 40.8%. Disease control rate at 1, 3, and 5 years was 89.7, 68.9, and 59.3%. Distant control rate at 1, 3, and 5 years was 97, 82.9, and 72.2%. Lung cancer‐specific survival at 1, 3, and 5 years was 95.6, 85.6, and 85.6%. Local control rate at 1, 3, and 5 years was 97.1%, 92.5, and 92.5%.

**FIGURE 2:**
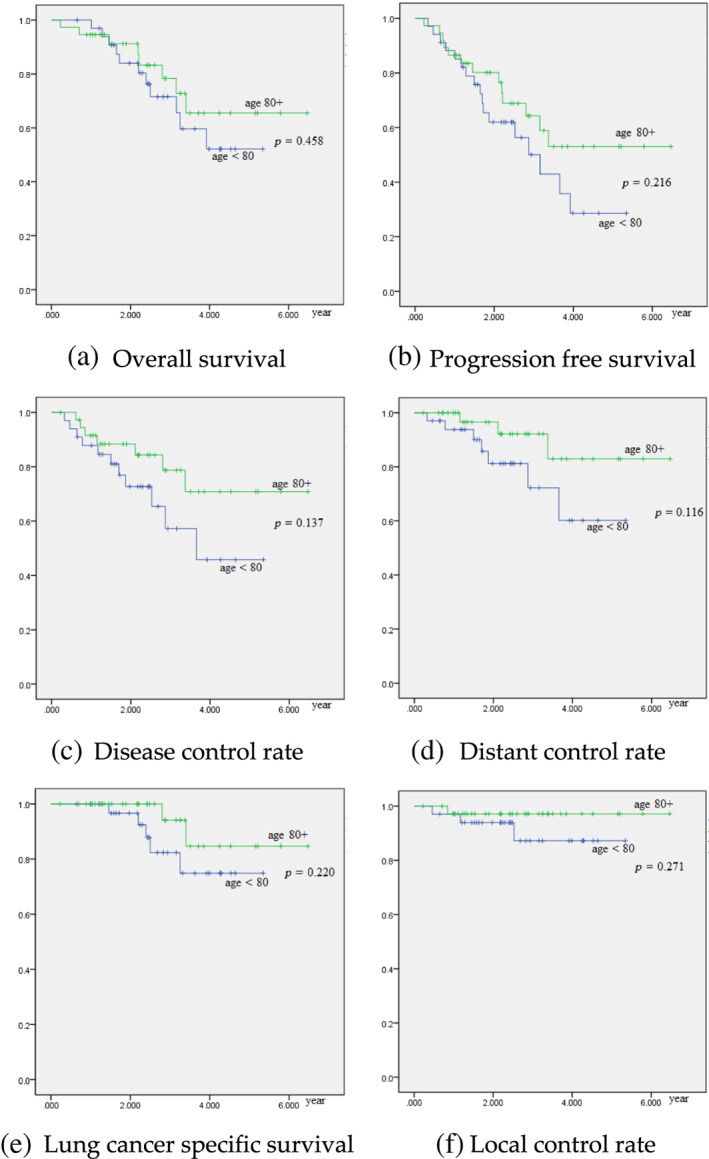
Univariate analysis on age 80 or more. (a) Overall survival, (b) progression‐free survival, (c) disease control rate, (d) distant control rate, (e) lung cancer specific survival and (f) local control rate

We compared the treatment outcomes of patients aged 80 or more to patients less than 80 years old and found no significant differences in overall survival (*p* = 0.458), progression‐free survival (*p* = 0.216), disease control rate (*p* = 0.137), distant control rate (*p* = 0.116), lung cancer‐specific survival (*p* = 0.220), and local control rate (*p* = 0.217) (Figure 2).

**FIGURE 3:**
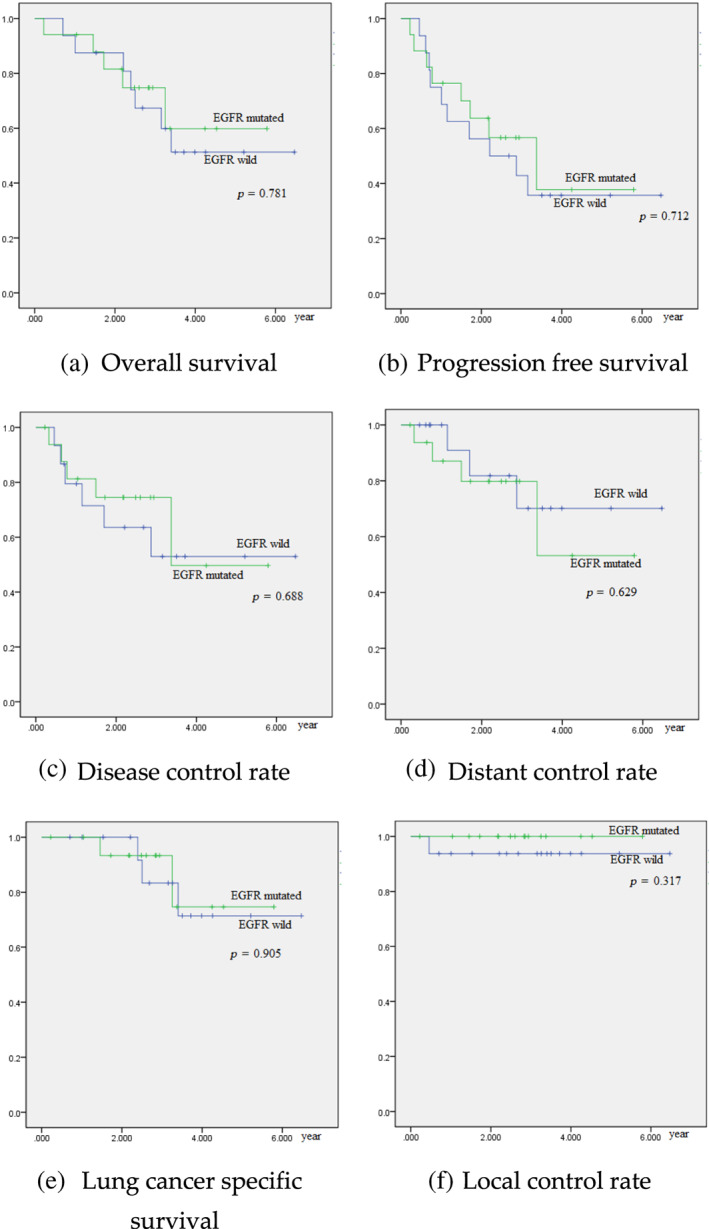
Univariate analysis on *EGFR* mutation: (a) overall survival, (b) progression‐free survival, (c) disease control rate, (d) distant control rate, (e) lung cancer specific survival and (f) local control

Among patients with EGFR status, there were no significant differences between patients with mutated *EGFR* and those with *EGFR* wild‐type in terms of overall survival (*p* = 0.781), progression‐free survival (*p* = 0.712), disease control rate (*p* = 0.688), distant control rate (p = 0.629), lung cancer‐specific survival (*p* = 0.905), or local control rate (*p* = 0.317) (Figure 3).

Because EGFR status and squamous cell carcinoma are highly colinear, we used Cox proportional regression with pathology type as a categorical variable for overall survival analysis.

Cox proportional regression on overall survival showed that there was a significant difference in sex, with females showing better overall survival than men (hazard ratio = 4.613, *p* = 0.041). Other factors, such as smoking status, T stage, recurrence, age over 80 years old, synchronous with other cancer, EGFR status, and pathological type, did not indicate significant differences in overall survival (Table 4).

**TABLE 4:** Cox proportional regression on overall survival

Risk factor	*p*‐value	Hazard ratio	95% confidence interval	
			Lower limit	Upper limit
Sex	0.041	4.613	1.062	20.029
Smoking	0.851	1.130	0.318	4.017
T stage	0.550	1.366	0.491	3.798
Recurrence	0.773	1.173	0.396	3.469
Age over 80 years old	0.359	0.623	0.227	1.712
synchronous cancer	0.416	1.748	0.456	6.704
Adenocarcinoma c/unknown EGFR	0.365	n/a	n/a	n/a
Adenocarcinoma c/*EGFR* mutation	0.277	0.273	0.026	2.833
Adenocarcinoma c/*EGFR* wild	0.977	1.035	0.101	10.653
Squamous cell carcinoma	0.834	0.773	0.070	8.556
others: one adenosquamous carcinoma and one NSCLC	0.296	0.252	0.019	3.352

Abbreviations: EGFR, epidermal growth factor receptor status; n/a, not available; NSCLC, non‐small cell lung cancer.

Cox proportional regression on progression‐free survival, disease control rate, distant control rate, lung cancer‐specific survival, or local control did not find significant risk factors.

### Toxicity

In 34 patients aged less than 80, there were four with grade 1 pneumonitis (defined by CTCAE vs. 4.03 as asymptomatic; clinical or diagnostic observations only; intervention not indicated). There were no patients with toxicity grade 2 or more in the cohort. In 37 patients aged 80 or more, there were eight with grade 1 pneumonitis and one patient with grade 1 malaise (defined by CTCAE vs. 4.03 as uneasiness or lack of wellbeing). Two patients were noted with grade 2 pneumonitis (defined by CTCAE vs. 4.03 as symptomatic; medical intervention indicated; limiting instrumental ADL). There was no grade 3 or more toxicity in the cohort.

Using a 2 x 3 chi‐square test among the two cohorts, we did not find a significant difference in toxicity (*p* = 0.1294).

The toxicities profile by *EGFR* mutation status are shown in Table 5. There was no significant difference among *EGFR* mutation status noted using a 2 x 3 chi‐square test (*p* = 0.322).

**TABLE 5:** Toxicity by *EGFR* mutation status

Toxicity	Grade 0	Grade 1	Grade 2
EGFR (+)	12	5	0
EGFR (−)	10	4	2

Abbreviation: EGFR, epidermal growth factor receptor status.

## DISCUSSION

In this study, we reviewed 71 consecutive patients at TVGH to investigate risk factors affecting oncological outcomes and toxicities for inoperable early‐stage NSCLC patients receiving SABR, including age ≧ 80 and *EGFR* mutation status. A previous study on 772 elderly patients did not show evident differences in progression‐free survival, lung cancer‐specific survival, or toxicity in patients aged 75 or more.^14^ Another study on 197 patients did not show an evident difference in treatment outcomes and toxicity between elderly (75–85 years old) and very elderly patients (>85 years old).^15^ However, one study on 335 patients found that age >75 years old was a risk factor for worse survival after adjusting with the Charlson comorbidity index (CCI).^16^ SABR has become essential in treating elderly patients with early‐stage NSCLC. For patients aged 75 or more, the population‐based in Netherland noted SABR introduction was associated with a 16% absolute increase in radiotherapy use, a decline in the proportion of untreated elderly patients, and an improvement in overall survival.^17^ For age ≧ 80, we also did not find any significant difference in oncological outcomes and toxicities in the current study. While whether old age would affect outcomes remains controversial,^14^, ^15^, ^16^ the current study could provide some evidence in treating patients age ≧ 80. Although Nakamura et al. found a higher out‐of‐field progression rate for *EGFR*‐mutant patients receiving SABR,^10^ in this cohort, we did not find a significant difference in disease control and local control rate across *EGFR* mutation status.

Males have been noted to have a shorter average life expectancy in the general population. As announced by the government, the average life expectancy in Taipei City for age 80 is 10.09 years and 12.34 years for males and females, respectively.^18^ The difference of sex on overall survival noted in the current cohort could be contributed by the intrinsic difference of the general population. Similarly, female gender has been noted as the only favorable variable on overall survival in another study on patients with potentially operable stage I NSCLC receiving SABR.^19^

The overall survival rate of early‐stage NSCLC patients receiving SABR is highly affected by comorbidities and general condition. For operable patients enrolled in the revised STARS trial, 5‐year OS has been reported as 87%.^20^ Another study using a population‐based database of Taiwan found that nonoperated NSCLC receiving SABR had 5‐year OS of only 31%.^21^ The 5‐year OS of 58.3% noted in this study is between the previous two studies and may reveal the current situation of a medical center in Taiwan. The local recurrence rate of 7.5% at 5‐years in the current study is comparable to the 6.3% noted in the revised STARS trial.^20^

In this study, we found a distant control rate of only 72.2% at 5 years. Effective systemic treatment is needed to prevent distant metastasis. For surgical patients, adjuvant osimertinib has shown survival benefits in *EGFR*‐mutated stage IB–IIIA patients in the ADAURA trial.^22^ For SABR patients with *EGFR* mutation, adjuvant osimertinib may also improve survival after SABR. Since no significant difference in survival was noted based on *EGFR* mutation status in this study, an improved prognosis may be noted with adjuvant osimertinib in patients with *EGFR* mutation.

There were some strengths in this study. With *EGFR* mutation status available in 33 (46.5%) patients, to the best of our knowledge, we analyzed the second largest cohort of *EGFR*‐mutated early‐stage NSCLC patients receiving SABR currently available in the literature. Additionally, the BED of SABR was relatively uniform, with 67 (94.3%) patients receiving BED10 ≥ 100 Gy, while the dose coverage has previously been noted to be associated with better local control and survival.^23^

Our study had a number of limitations. First, it was a retrospective analysis and there may have been some selection bias. Second, because the patient group was not large enough, our study may not have enough statistical power to analyze the impact of other factors. Third, because the dose‐fractionation was tailored to their clinical condition, the prescribed radiation dose was not uniform.

In conclusion, in this study we reviewed 71 patients with inoperable early‐stage non‐small cell lung cancer receiving SABR at TVGH. Male gender was noted as a risk factor for poor overall survival. No significant differences were noted in treatment outcomes or toxicity for patients age ≧80 or with *EGFR* mutation.

## AUTHOR CONTRIBUTIONS

Conceptualization, Yuan‐Hung Wu; methodology, Yuan‐Hung Wu; software, Yu‐Wen Hu; validation, Yu‐Mei Kang, Yu‐Wen Hu, Heng‐sheng Chao, Chi‐Lu Chiang; formal analysis, Yuan‐Hung Wu, Yu‐Mei Kang, Yu‐Wen Hu; resources, Yuan‐Hung Wu, Yu‐Mei Kang, Yu‐Wen Hu, Keng‐Li Lan, Sang‐Hue Yen, Tzu‐Yu Lai, Tien‐Li Lan, Yuh‐Min Chen, Chao‐Hua Chiu, Yung‐Hung Luo, Heng‐sheng Chao, Chi‐Lu Chiang, Tsu‐Hui Shiao, Chao‐Neng Yang, Wen‐Hu Hsu, Yu‐Chung Wu, Han‐Shui Hsu, Jung‐Jyh Hung, Chien‐Sheng Huang, Po‐Kuei Hsu, Yi‐Wei Chen; writing—original draft preparation, Yuan‐Hung Wu; writing—review and editing, Yuan‐Hung Wu, Yu‐Mei Kang; supervision, Yi‐Wei Chen. All authors have read and agreed to the published version of the manuscript.

## FUNDING INFORMATION

This research received no funding.

## CONFLICT OF INTEREST

The authors declare no conflict of interest.
